# Complete Genome Sequence of Pseudomonas Phage Motto

**DOI:** 10.1128/mra.00740-22

**Published:** 2022-10-12

**Authors:** Prasanth Manohar, Archana Loganathan, Ramesh Nachimuthu, Belinda Loh, Long Ma, Dann Turner, Sebastian Leptihn

**Affiliations:** a Zhejiang University-University of Edinburgh (ZJE-UoE) Institute, Zhejiang University School of Medicine, Haining, People’s Republic of China; b School of Bioscience and Technology, Vellore Institute of Technology (VIT), Vellore, Tamil Nadu, India; c Fraunhofer Institute for Cell Therapy and Immunology (IZI), Department of Antimicrobial Biotechnology, Leipzig, Germany; d School of Applied Sciences, College of Health, Science and Society, University of the West of England, Bristol, United Kingdom; Queens College CUNY

## Abstract

We describe the complete genome sequence of bacteriophage Motto, which infects clinical strains of Pseudomonas aeruginosa. Motto is a T1-like siphovirus related to members of the family *Drexlerviridae* and has a capsid width of ~57 nm and a tail length of ~255 nm. The 49.9-kb genome contains 84 protein-coding genes.

## ANNOUNCEMENT

Pseudomonas aeruginosa is a Gram-negative pathogen that causes life-threatening infections in humans, especially associated with ventilators and surgical wounds (1, 2). P. aeruginosa continues to acquire molecular determinants to avoid the detrimental effects of antibiotics, and infections caused by such multidrug-resistant strains are difficult to treat (3). Phage therapy, the use of bacteriophages as therapeutic agents, offer a new hope to treat antibiotic-resistant infections (3–5). Here, we present the genome of the Pseudomonas phage Motto isolated from a water sample collected at the Cooum River in Chennai (13.0827°N, 80.2707°E), Tamil Nadu, India.

P. aeruginosa PAO1 was used as the host bacterium for the isolation and propagation of phage Motto. The phage enrichment method was used for bacteriophage isolation (6). Briefly, to 10 mL of exponentially grown bacterial (host) culture in Luria-Bertani (LB) broth, 30 mL of water sample was added, and the mixture was incubated at 37°C for 20 h. Then, the mixture was centrifuged at 6,000 × *g* for 15 min and the supernatant was collected. The collected phage lysate was filtered through 0.22-μm-pore syringe filters and tested for the presence of bacteriophage using a double-agar-overlay method (6). Phage purification was performed as described previously (6). Phage morphology was determined using transmission electron microscopy (TEM) after negative staining with 2% (wt/vol) uranyl acetate (Merck, Germany).

To isolate the genomic DNA of phage Motto, phenol-chloroform extraction was used (7). The Nextera XT DNA library preparation kit was used to create the sequencing libraries. The phage genome was then sequenced using the Illumina HiSeq platform. A total of 11,771,058 reads were produced, and the total number of read bases was 1.8 Gbp. A total of 5,885,529 clean reads of 150 bp (paired-end format) were used to assemble the phage genome, with 100% coverage and 7,800× depth. The short-read sequence data were assembled using Unicycler (v.0.4.7) (8). The assembly was performed after quality filtering and quality control employing FastQC, MultiQC, and Trimmomatic (9–11) and completion of the assembled genome was determined, and the coverage and depth were calculated by BEDTools (12). Genome annotation was completed using Prokka 1.14.5 (13) and Galaxy-Apollo (14). All tools were run with default parameters unless otherwise specified.

**FIG 1:**
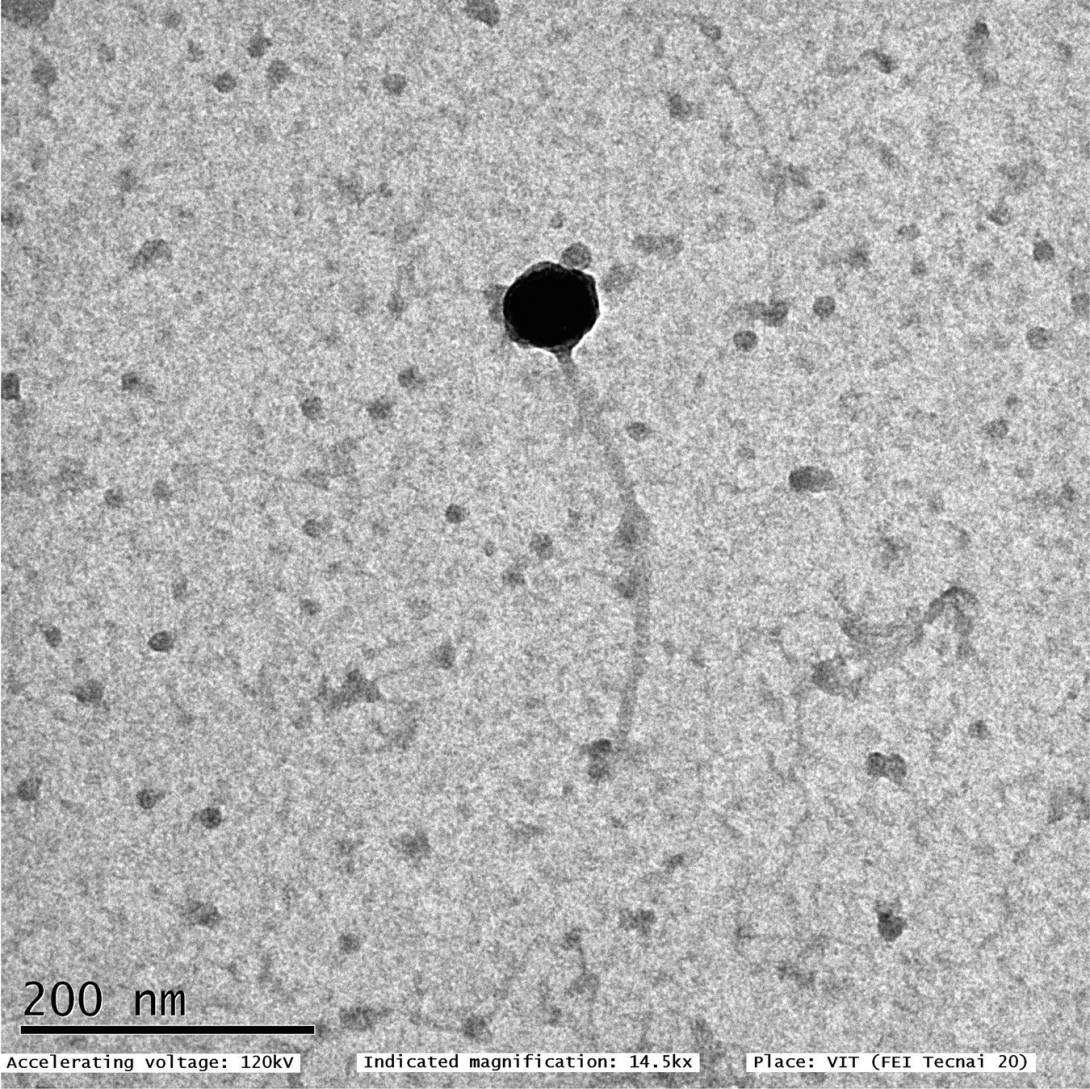
Transmission electron microscopic image of Pseudomonas phage Motto.

Pseudomonas phage Motto has an icosahedral head of about 57 ± 1 nm and a long noncontractile tail 255 ± 1.5 nm in length (Fig. 1). Based on its morphology, Motto appears to belong to the T1-like viruses in the order *Caudoviricetes*. Motto contains a double-stranded DNA (dsDNA) genome of 49,960 bp with a G+C content of 45%, with 84 predicted open reading frames and no tRNAs identified by ARAGORN (15). Blastn was used on the entire genome sequence, determining query coverage of both of the closest relatives of Motto that can be found in the NCBI database, *Vibrio* virus 2019VC1 (NC_054898.1; query coverage, 43%; sequence identity, 88%) and Salmonella virus STSR3 (MT500539.1; query coverage, 65%; sequence identity, 82%). Motto likely belongs to the *Drexlerviridae*, based on a preliminary analysis using blastn with the entire genome. Motto was characterized as lytic using PhageAI (16).

### Data availability.

The whole-genome sequencing data are available through NCBI Sequence Read Archive (BioProject accession number PRJNA882249; run number SRR21708404). The annotated genome assembly is available through NCBI GenBank under accession number ON843697.
